# Supplementary material for: Complexome profiling of the Chlamydomonas psb28 mutant reveals TEF5 as an early PSII assembly factor
Source: Plant Cell. 2025 Mar 17;37(6):koaf055. doi: 10.1093/plcell/koaf055 (PMC12164590; doi:10.1093/plcell/koaf055)
Supplement: koaf055_Supplementary_Data [file koaf055_supplementary_data.zip › psb28_interactive.zip!/plots/intensity/qualityPlots/peptides/THF1_Cre13.g562850.t1.2.html]

### Cre13.g562850.t1.2

Trivial name: THF1  
SeqCoverage [median]: 56.40  
Mass: 28.59 kDa  
  
 

| Sequence | MissedCleavage | Mass [Da] | wtIntensity | muIntensity | Score |
| --- | --- | --- | --- | --- | --- |
| AAGTESSPAQVQA | 0 | 1215.57 | 32925.52 | 55344.14 | 175.93 |
| ALASISSASAAGAFSYNK | 0 | 1714.85 | 597.32 | 0.00 | 191.48 |
| AVATPVLAATFKPPTVAETK | 1 | 2011.14 | 7109.73 | 24807.47 | 115.57 |
| AVGVKPEAVNR | 1 | 1138.65 | 50175.40 | 106638.14 | 136.50 |
| AVGVKPEAVNRDLLMYKGVLSK | 3 | 2386.34 | 0.00 | 3639.46 | 48.31 |
| AYVDALGEDADK | 0 | 1265.58 | 483.97 | 9080.89 | 175.86 |
| AYVDALGEDADKYK | 1 | 1556.74 | 1515.24 | 4303.73 | 108.59 |
| AYVDALGEDADKYKR | 2 | 1712.84 | 20186.77 | 39299.63 | 118.13 |
| DASALEQAANGLTPESLTPNADGNEVQK | 0 | 2839.35 | 9748.01 | 9178.69 | 112.26 |
| ELMREFVER | 1 | 1207.60 | 8528.19 | 10896.57 | 110.53 |
| ELMREFVEREK | 2 | 1464.74 | 2353.17 | 2999.25 | 143.00 |
| EPSALEK | 0 | 772.40 | 433.49 | 5249.07 | 73.98 |
| FVAIGLFR | 0 | 921.54 | 1309.78 | 19522.80 | 149.06 |
| LLELSGAK | 0 | 829.49 | 827.30 | 8556.78 | 109.01 |
| LLELSGAKEPSALEK | 1 | 1583.88 | 34301.51 | 104271.08 | 169.94 |
|  |  |  | 170495.4 | 403787.7 |  |
